# LSD1 inhibition yields functional insulin-producing cells from human embryonic stem cells

**DOI:** 10.1186/s13287-020-01674-y

**Published:** 2020-04-28

**Authors:** Fei He, Ning Li, Hai-Bo Huang, Jing-Bo Wang, Xiao-Fei Yang, Hua-Dong Wang, Wei Huang, Fu-Rong Li

**Affiliations:** 1 Translational Medicine Collaborative Innovation Center, The Second Clinical Medical College (Shenzhen People’s Hospital), Jinan University, 1017 Dongmen North Road, Shenzhen, 518020 China; 2 Integrated Chinese and Western Medicine Postdoctoral Research Station, Jinan University, Guangzhou, 510632 China; 3 Guangdong Engineering Technology Research Center of Stem Cell and Cell therapy, Shenzhen, 518020 China; 4 Shenzhen Key Laboratory of Stem Cell Research and Clinical Transformation, Shenzhen, 518020 China; 5 Department of Pathophysiology, Key Laboratory of State Administration of Traditional Chinese Medicine, School of Medicine, Jinan University, Guangzhou, 510632 China; 6 Department of Biology, Southern University of Science and Technology, Shenzhen, 518055 China

**Keywords:** Human embryonic stem cells, Differentiation, Insulin-producing cells, Lysine-specific demethylase 1(LSD1)

## Abstract

**Background:**

Human embryonic stem cells represent a potentially unlimited source of insulin-producing cells for diabetes therapy. While tremendous progress has been made in directed differentiation of human embryonic stem cells into IPCs in vitro, the mechanisms controlling its differentiation and function are not fully understood. Previous studies revealed that lysine-specific demethylase 1(LSD1) balanced the self-renewal and differentiation in human induced pluripotent stem cells and human embryonic stem cells. This study aims to explore the role of LSD1 in directed differentiation of human embryonic stem cells into insulin-producing cells.

**Methods:**

Human embryonic stem cell line H9 was induced into insulin-producing cells by a four-step differentiation protocol. Lentivirus transfection was applied to knockdown LSD1 expression. Immunofluorescence assay and flow cytometry were utilized to check differentiation efficiency. Western blot was used to examine signaling pathway proteins and differentiation-associated proteins. Insulin/C-peptide release was assayed by ELISA. Statistical analysis between groups was carried out with one-way ANOVA tests or a student’s *t* test when appropriate.

**Results:**

Inhibition or silencing LSD1 promotes the specification of pancreatic progenitors and finally the commitment of functional insulin-producing β cells; Moreover, inhibition or silencing LSD1 activated ERK signaling and upregulated pancreatic progenitor associated genes, accelerating pre-maturation of pancreatic progenitors, and conferred the NKX6.1^+^ population with better proliferation ability. IPCs with LSD1 inhibitor tranylcypromine treatment displayed enhanced insulin secretion in response to glucose stimulation.

**Conclusions:**

We identify a novel role of LSD1 inhibition in promoting IPCs differentiation from hESCs, which would be emerged as potential intervention for generation of functional pancreatic β cells to cure diabetes.

## Introduction

Diabetes mellitus is a chronic metabolic syndrome featured by elevated blood glucose levels results from inadequate production of the hormone insulin. It was estimated over 451 million adults have diabetes, and diabetes has been a major health problem worldwide [1]. Patients with diabetes are usually coupled with insulin reduction results from pancreatic β-cell deficiency or substantial β-cell loss [2]. Currently, the most popular treatment is daily insulin injections, but it is demanding and prone to complications because it is impossible to mimic the insulin dynamic changes in vivo. A perfect solution to diabetes would be to replenish the mass of functional pancreatic beta cells (insulin-producing cells, IPCs) [3]. Human embryonic stem cells (hESCs) represent an attractive source for β-cells production, owing to its pluripotency and unlimited proliferation features [4]. Several protocols to differentiate human embryonic stem cells and induced pluripotent stem cells (iPSCs) into human pancreatic β-like cells have been developed [5–9]. Although marked progress has been made in identifying signaling pathways or key regulators during producing pancreatic endocrine cells from hESCs, the challenge of generating fully matured β cells remains. Finding out a powerful regulator and optimizing the differentiation protocols to largely enhance IPCs production seems to be an urgent need in this field [10, 11].

The generation of different cell types from stem cells is epigenetically regulated and the factors involved in these processes are often essential for development [12]. Major epigenetic events, such as DNA methylation, modification of histones, and non-coding RNAs expression, orchestrate physiological endocrine pancreas specification into β-like cells, both in vivo and in vitro. Understanding the epigenomic modifiers underlying endocrine pancreas development could, therefore, improve in vitro differentiation methods [13]. LSD1 (histone lysine-specific demethylase 1; also known as KDM1A) was initially identified as a transcriptional repressor via demethylation of active histone H3 marks including monomethyl lysine 4 (H3K4me1) and di-methyl lysine 4 (H3K4me2) [14]. LSD1 is a key histone modifier that maintains the pluripotency of hESCs through demethylation of histone H3 lysine 4 (H3K4) and subsequent repression of genes controlling cell differentiation [15]. Our previous study confirmed its vital role in balancing self-renewal and differentiation of human induced pluripotent stem cells (hiPSCs), and hiPSCs is converted to differentiation from proliferation when LSD1 activity was at approximately 50% [16]. LSD1 is essential in decommissioning enhancers during the differentiation of mouse embryonic stem cells (mESCs) [17]. LSD1 regulates differentiation onset of trophoblast stem cells (TSCs) by repressing transcription factor Ovol2 in undifferentiated cells [18]. Moreover, LSD1 is widely involved in stem cell differentiation process including neural stem cells differentiation [19, 20], adipogenic differentiation [21, 22], and myogenic differentiation [23]. However, the role of LSD1 in pancreatic differentiation is not fully understood.

In the present study, we adopted a four-step differentiation protocol to generate IPCs from hESC H9 and used shRNA lentivirus to knockdown LSD1 expression, TCP, to inhibit its activity. We demonstrated that the expression level of LSD1 mRNA and protein was gradually reduced during IPCs differentiation from hESCs. Besides, we show that inhibition or depletion of LSD1 promotes hESCs differentiation into pancreatic progenitors and then IPCs in vitro, by activating ERK signaling and upregulating pancreatic progenitors associated genes. Sustained treatment of LSD1 inhibitor TCP helps IPCs maturation witnessed by improved glucose-stimulated insulin secretion (GSIS). Our data may cast light on the role of LSD1 in the process of IPC differentiation from hESCs and makes it possible for manufacturing functional IPCs, which would emerge as a candidate cell therapy product of diabetes.

## Materials and methods

### Culturing and differentiation of human ES cells into pancreatic β cells

Human embryonic stem cell line H9 was obtained from National Stem Cell Bank c/o WiCell Research Institute. H9 cells were cultured in a six-well cell culture microplate (Corning, #3516) coated with Matrigel (Corning, #354277) and in mTeSR™1 medium (Stem Cell Technologies, #85850). For differentiation, H9 cells were dissociated into small clumps by dispase (Invitrogen, #12604-021, 4 min at 37 °C) and then plated into a Matrigel-coated six-well plate for attachment. The differentiation process was started when H9 cells expanded to a 60% coverage of the well.

Stage 1: Day 1, DMEM/F12 supplemented with 0.2% BSA (Sigma, A1933) containing 100 ng/ml activin A (Peprotech, AF-120-14E) and 1 μM Wnt 3a (R&D systems, 5036-WN-010). Days 2–4: DMEM/F12 (supplemented with 0.2% BSA) containing 100 ng/ml activin A.

Stage 2: The differentiated cells were cultured in F12/IMDM (1:1, supplemented with 0.5% BSA, 0.5% ITS and 0.5× B27) with 50 ng/ml NOGGIN (Peprotech, 120-10C), 20 ng/ml FGF7 (Peprotech, AF-100-19), and 2 μM RA (Sigma, R7882) for 4 days.

Stage 3: The cells were cultured in DMEM (high glucose; supplemented with 0.5% BSA, 1% ITS and 1× N2) with 50 ng/ml EGF (Sigma, E9644) for 5 days, and the cells exhibited obvious expansion and reached nearly 100% coverage.

Stage 4: For maturation, another 7–9 days incubation in DF12 media with 1% ITS, 10 ng/ml bFGF (Peprotech, AF-100-18B), 10 mM nicotinamide (Sigma, N0636), 50 ng/ml Exendin-4 (Sigma, E7144), and 10 ng/ml BMP4 (Peprotech, AF-120-05E) for maturation was performed. All media were purchased from Gibco.

### LSD1 inhibitor treatment and LSD1 activity assay

LSD1 inhibitor Tranylcypromine 1/2H_2_SO_4_ (TCP, Merck, 13492-01-8) was dissolved in ddH2O. Concentration of TCP was 40.0 μM based on our previous study and inhibition curve we obtained. The medium was replaced with fresh medium containing the drug daily. Nuclear extract/LSD1 were prepared using the Epi-Quik™ Nuclear Extraction Kit II (Epigentek, OP-0022-100) and LSD1 activity were tested by the EpiQuik™ Histone Demethylase LSD1 Activity/Inhibition Assay Kit (Epigentek, P-3079-96) following the manufacturer’s instructions, respectively.

### Lentivirus-mediated transfection

LSD1 shRNA plasmids and lentiviral particles were purchase from Shanghai Genechem Co. Ltd. produced in 293 T cells. The lentiviral vectors used in this study were GV248 (hU6-MCS-Ubiquitin-EGFP-IRES-puromycin). Inserted sequences of sh1, sh2, sh3, and control shRNA were listed in Additional file: Table S1. After 24 h attachment, cells were infected by lentiviral particles at the concentration of MOI = 20 in the stage-matched medium with polybrene reagent (Merck, H9268) at 5 μg/mL. Differentiation process was started 48 h later, and cells were harvested at different stages according to the differentiation protocol for different analyses.

### RNA extraction and quantitative PCR analysis of gene expression

Total RNA was isolated using TRIzol® Reagent (Life Technologies, 15596-018) according to manufacturers’ instructions. Subsequently, complementary DNA was generated using the PrimeScript™ RT reagent Kit with gDNA eraser (Takara Bio Inc., RR047A), and quantitative real-time reverse transcription polymerase chain reaction (qPCR) was performed using the Real-Time PCR detection system (Applied Biosystems, 7500) with 2× SYBR Green II/ROX qPCR Master Mix (Takara Bio Inc., RR82LR). Relative mRNA expression was calculated using the delta threshold cycle (ΔΔCT) method and normalized to β-actin (ACTB) expression. The PCR primers are listed in Additional file: Table S2.

### Immunofluorescence assay

Cells grown in 6-well plates were fixed for 15 min in 4% (w/v) paraformaldehyde at room temperature, washed 3 times with PBS, and treated with 0.1% (v/v) Triton X-100 (Sigma-Aldrich, T9284) for 15 min and then blocked for 60 min with 5% (v/v) normal serum in PBS. After that, cells were incubated with primary antibodies at 4 °C overnight and secondary antibodies for 1 h at room temperature, followed by washing and staining with DAPI (2 μg/ml, Sigma-Aldrich, D8417). The following primary antibodies used in this study were listed in Additional file: Table S3. Alexa Fluro secondary antibodies from Molecular Probes were used at 1:1000. Images were taken using a Leica microscope (Leica, DMi8) with its software LAS X.

### Flow cytometry

Differentiated cell clusters or islets were digested into single-cell suspension by incubation in TrypLE (Thermo Scientific, 12,563,029) at 37 °C (typically 7–10 min). The TrypLE was quenched with 3–4 volumes of DMEM/F12 (1:1) media and cells were centrifuged for 5 min at 300×*g*. Before transferred into a 1.5-ml microtube (Axygen, MCT-150-C), cells were washed once in PBS (1 mL). Immunostaining for FACS analysis using anti-SOX17, CXCR4, PDX1, NKX6.1 SOX9, C-peptide, and insulin antibodies was carried out using the Fixation/Permeabilization Solution Kit (BD Biosciences, 554714) from BD according to the manufacturer’s instructions. In short, cells were finally resuspended in 300 μl sorting buffer and filtered into 1.5-ml microtubes, and analyzed by Beckman Coulter flow cytometer (Beckman Coulter, DxFLEX) with at least 20,000 events recorded for each test. Analysis of the results was performed using FlowJo software.

### Western blot

Total protein was extracted from cells in RIPA lysis buffer (Beyotime, P0013B) and quantified using a Bradford assay. In total, 30 μg of protein was separated using 10% sodium dodecyl sulfate-polyacrylamide gel electrophoresis (SDS-PAGE) and then transferred to a polyvinylidene difluoride (PVDF) membrane (Millipore, ISEQ0010). The membrane was blocked in a 5% powdered milk solution and incubated in primary antibody overnight at 4 °C. After washing, the membrane was incubated with a horseradish peroxidase–conjugated secondary antibody at 37 °C for 1 h. Protein bands were visualized using Western Bright ECL (Millipore, WBKLS0500) and detected using ImageQuant LAS4000mini (General Electric, USA). Relative protein levels were calculated based on a β-Actin loading control. Antibodies used for Western blot were listed in Additional file: Table S3.

### Insulin/C-peptide release assay by ELISA

After 22 days of differentiation, approximately 8 × 10^5^ insulin-producing cells in clusters each well were washed with Krebs buffer and incubated in low (2.8 mM) glucose Krebs in cell culture inserts (Millicell) to starve cells and remove residual insulin at 37 °C for 1 h. Clusters were washed and incubated in low glucose Krebs for 1 h and the supernatant was collected. They were then transferred to high (20 mM) glucose, incubated for 1 h and the supernatant was collected. For sequential GSIS challenges, this sequence of low- and high-glucose stimulations was repeated twice. At the end, clusters were incubated in 2.8 mM glucose + 30 mM KCl Krebs (depolarization challenge) for 1 h and the supernatant was collected. Clusters were dispersed into single cells using TrypLE and cell number was estimated by a Vi-Cell counter (Beckman Coulter). Insulin concentration was determined for supernatant samples using the Human Ultrasensitive Insulin ELISA (ALPCO Diagnostics). Protein extraction was performed with the M-PER extraction reagent (Thermo Scientific, 78501), and insulin content was measured for each sample using the human Ultrasensitive Insulin ELISA kit. Insulin secretion levels were normalized to total insulin content for each sample. Stimulation indexes were calculated as a ratio of insulin secretion at high glucose (20 mM) relative to the basal secretion (2.8 mM glucose).

### Statistical analyses

Data are expressed as mean ± SEM. Statistical analysis was assessed by GraphPad Prism 7 for Windows. Comparisons between groups were carried out with one-way ANOVA tests or a student’s *t* test when appropriate. A *p* value < 0.05 was considered statistically significant.

## Results

### LSD1 is downregulated during pancreatic β cells differentiation

**Fig. 1:**
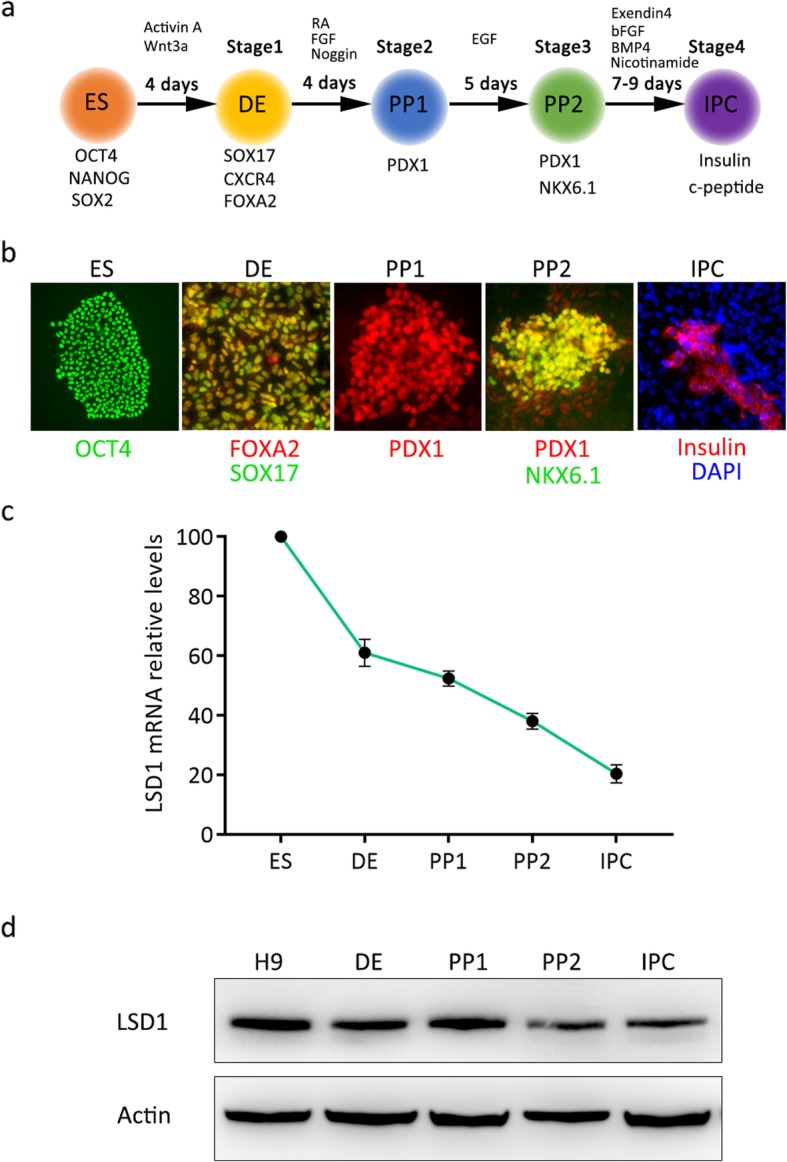
LSD1 expression changes during human embryonic stem cell differentiation into insulin-producing β cells. **a** Schematic of a step-wise differentiation protocol of hESCs into insulin-producing β cells. ES, embryonic stem cell; DE, definitive endoderm cells; PP1, PDX1^+^NKX6.1^−^ pancreatic progenitor cells; PP2, PDX1^+^NKX6.1^+^ pancreatic progenitor cells; IPC, insulin-producing cells. **b** Representative immunofluorescence images stained with marker genes of different stages. **c** Relative mRNA expression of LSD1 at different differentiation stages. **d** Representative Western blot images of LSD1 protein level at the different differentiation stages

In this study, we used a modified, highly efficient step-wise protocol, which was previously developed by Deng’s group [6], to direct pancreatic differentiation from the human embryonic stem cell line H9 (Fig. 1a). First, Activin A and Wnt 3a were utilized to induce definitive endoderm (DE) formation for 4 days. Secondly, RA, FGF, and Noggin were used for PP1 formation for 4 days. Furthermore, EGF was applied to generate PP2 cells for 5 days. Lastly, a combination of Exendin 4, bFGF, BMP4, and Nicotinamide induced PP2 cells into IPCs in 7–9 days. The marker genes of different differentiation stages are listed below the schematic diagram (Fig. 1a). Representative cell images of ES, DE, PP1, PP2, and IPCs stained with their marker genes are shown in Fig. 1b. We successfully obtained insulin-producing cells at the end of the differentiation process. LSD1 expression was examined during the multistep directed differentiation of hESCs into IPCs as outlined in Fig. 1a. During the IPC differentiation of hESCs, relative mRNA levels of LSD1 decreased gradually, which was shown in Fig. 1c. Besides, we observed LSD1 protein expression throughout stage 1 to stage 4, and found that expression of LSD1 began to decrease at the very beginning of differentiation, which was consistent with previous studies [15, 18] as shown in Fig. 1d. In all, downregulation of LSD1 happened during the differentiation process of insulin-producing cells from hESCs. Given that LSD1 expression was gradually reduced during pancreatic development, we carried out the following inactivation experiments to investigate whether LSD1 played a role in the IPC differentiation of hESCs and in which stage LSD1 was most effective.

### Knockdown or inhibition of LSD1 does not affect the specification of DEs

**Fig. 2:**
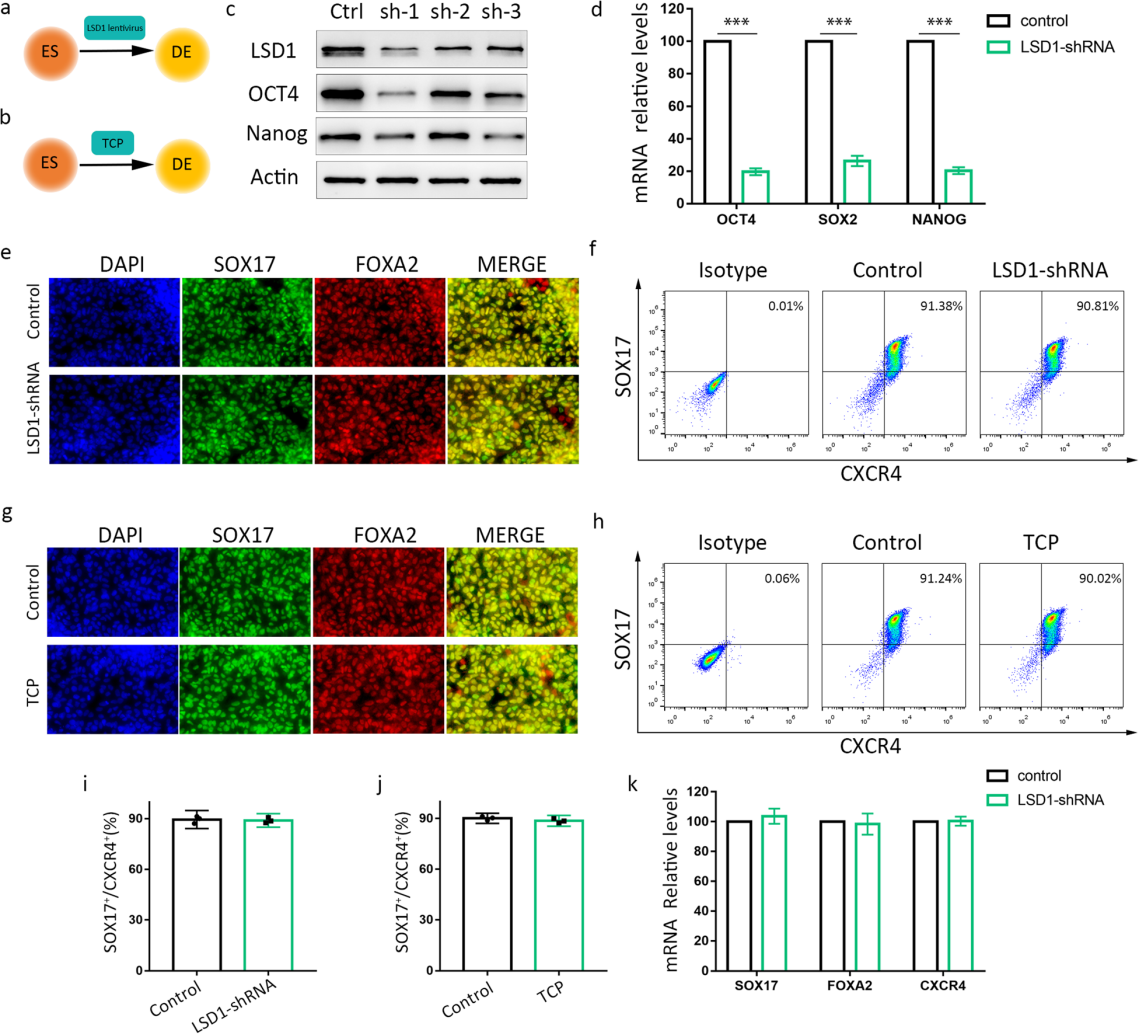
Effect of LSD1 knockdown or inhibition on hESCs differentiation into DE cells. **a** Diagram of experimental design for **c**–**f**, **i**, and **k**. **b** Diagram of experimental design for **g**, **h**, and **j**. **c** Protein levels of LSD1, oct4, and Nanog detected by Western blot at the end of DE **d** Relative mRNA expression of pluripotency genes COT4, SOX2, and Nanog in ES treated with LSD1 shRNAs. **e** The co-expression of SOX17 with FOXA2 was detected by immunofluorescence assay at the definitive endoderm stage in control and LSD1-shRNA group (**e**), control and TCP group (**g**), respectively. **f**, **h**–**j** Effects of LSD1 shRNAs (**f**) and TCP (**h**) during definitive endoderm differentiation on SOX17 and CXCR4 expression and quantification of the proportion of SOX17^+^/CXCR4^+^ (**i**, **j**) by flow cytometry. **k** The mRNA expression of DE-associated genes SOX17, FOXA2, and CXCR4 in control and LSD1 shRNA groups. Data represent mean ± SEM, *p < 0.05, **p < 0.01, ***p < 0.001, two-sided student’s *t* test (*n* = 3 biologically independent samples per group)

To determine the role of LSD1 in the process of IPC differentiation from hESCs, LSD1 was silenced and inhibited, respectively. The experimental designs depicting the time points of lentiviral or tranylcypromine (TCP) intervention initiation and sample collection were presented in Fig. 2a, b. Three LSD1-targeting shRNAs were used, and sh-1 was the most effective for LSD1 knockdown, which was thus utilized in the following experiments. With LSD1 protein expression decreasing, the protein levels of the pluripotency genes including OCT4 and Nanog were reduced accordingly (Fig. 2c). Moreover, the relative mRNA levels of OCT4, SOX2, and Nanog were reduced by more than 70% (Fig. 2d) by the end of definitive endoderm differentiation. Accordingly, the changes in cell morphology were shown in Fig. S1a after 2-day lentiviral infection, in which GFP showed transfection efficiency of shRNA lentivirus.

To explore the possible effect of the LSD1 shRNA lentivirus on DE differentiation of hESCs, immunostaining assay of cell clusters was applied to show the co-expression of DE marker genes SOX17 and FOXA2 in Fig. 2e, and flow cytometry to check the ratio of SOX17^+^CXCR4^+^ cells (Fig. 2f, i). Both immunofluorescence assay and flow cytometry analysis showed no significant difference between control and LSD1 shRNA group in DE differentiation efficiency, as the ratio of the cells stained with double marker genes accounts for nearly 90%. Besides, the mRNA relative levels of SOX17, FOXA2, and CXCR4 were not significantly changed after LSD1 inhibition (Fig. 2k) at the end of DE differentiation. To further confirm our results, we used a monoamine oxidase inhibitor tranylcypromine (TCP) to inhibit LSD1 activity in hESCs. Firstly, we made an inhibition curve of LSD1 activity by TCP in hESCs and selected an optimum dose which was 40 μM with inhibition ratio of 65% (Fig. S1b, S1c). Most importantly, in agreement with the finding from LSD1 silencing, inhibition of LSD1 by TCP in the stage 1 cells sustained the proportion of SOX17^+^CXCR4^+^ cells compared with the control group (Fig. 2g, h, j). So, it could be concluded that LSD1 does not affect DE differentiation from hESCs.

### Silencing or inhibition LSD1 promotes the specification of PP2

**Fig. 3:**
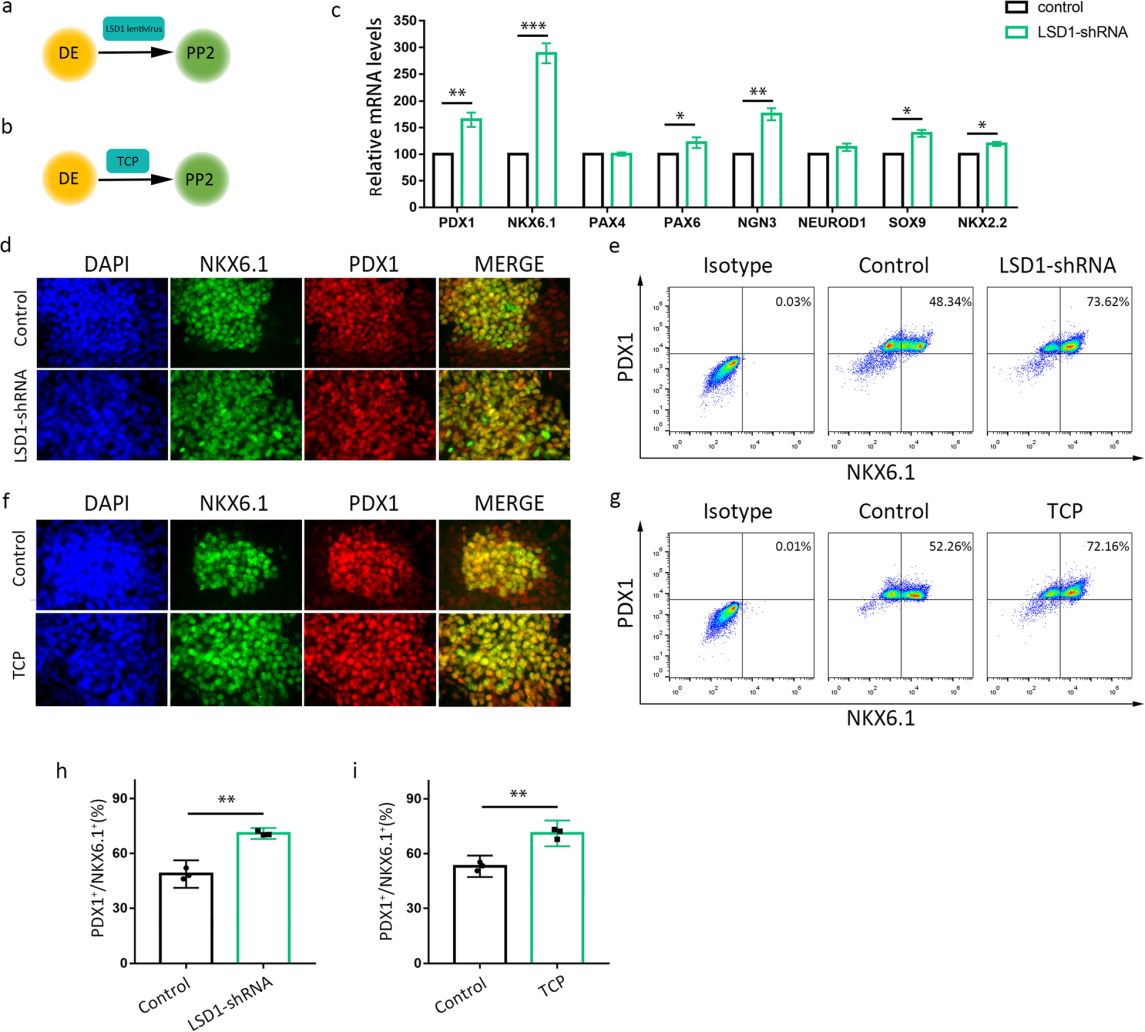
LSD1 knockdown or inhibition enhances the generation of PP2 cells. **a** Diagram of experimental design for **d**, **e**, and **h**. **b** Diagram of experimental design for **f**, **g**, and **i**. **c** Relative mRNA expression of pancreatic progenitor associated genes detected by real-time PCR at the end of PP2 after LSD1 knockdown (**d**, **f**). The co-expression of PDX1 with NKX6.1 was detected by immunofluorescence assay at stage 3 in control and LSD1-shRNA group (**d**) and control and TCP group (**f**), respectively. Effects of LSD1 shRNAs (**e**, **h**) and TCP (**g**, **i**) on the co-expression of PDX1 and NKX6.1 during pancreatic progenitor differentiation and quantification of the proportion of PDX1^+^/NKX6.1^+^, respectively. Data represent mean ± SEM, *p < 0.05, **p < 0.01, ***p < 0.001, two-sided student’s *t* test (*n* = 3 biologically independent samples per group)

We sought to determine whether LSD1 regulates pancreatic progenitor differentiation by treating cells with LSD1 shRNA lentivirus or its inhibitor TCP (Fig. 3a, b). When LSD1 shRNA lentivirus is added during the specification of stage 1 (DE) to stage 3 (PP2), immunostaining results shows a dramatic increase in the proportion of PP2 cells co-expressing PDX1 and NKX6.1 (Fig. 3d). The treatment results in a significant increase in the proportion of PDX1^+^NKX6.1^+^ progenitors compared with control groups (48.34% vs 73.62%) detected by flow cytometry (Fig. 3e, h). The mRNA abundance of the transcription factors critical for pancreatic progenitor cell differentiation, including PDX1, NKX6.1, PAX6, NGN3, SOX9, and NKX2.2, was significantly increased after LSD1 knockdown in the PP2 cells (pancreatic progenitors). On the contrary, there was no significant change in the mRNA expression level of PAX4 and NEUROD1 (Fig. 3c). In agreement with this result, TCP treatment from DE to PP2 differentiation also yielded similar results, with a PDX1^+^NKX6.1^+^ progenitors ratio raised from 52.26 to 72.16% (Fig. 3f, g, i). In all, our study found that either silencing or inhibiting LSD1 strongly promotes hESCs differentiation into pancreatic progenitor cells.

### LSD1 modulates PP2 specification through ERK signaling

**Fig. 4:**
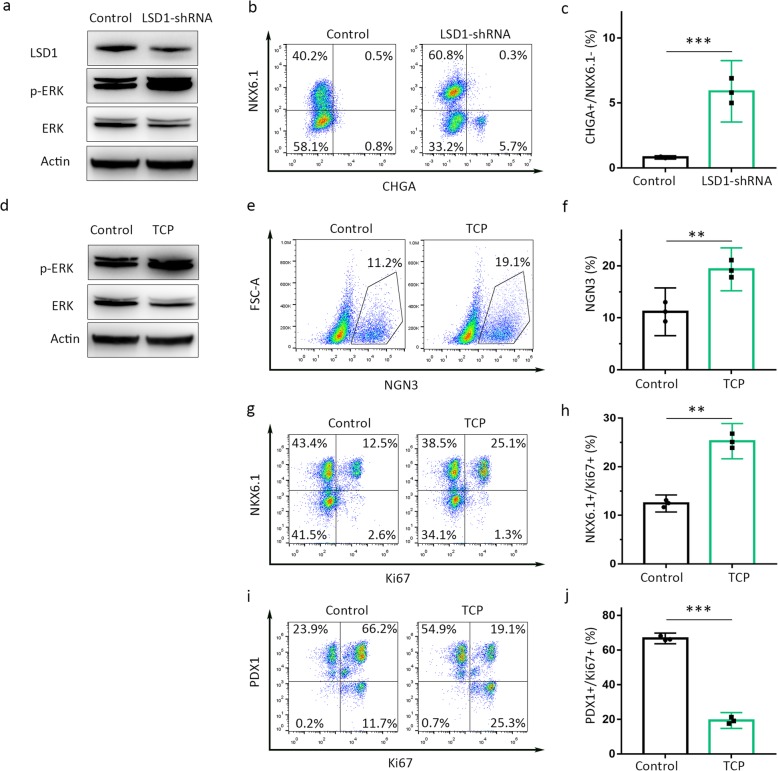
ERK signaling was activated during PP2 specification. **a** The protein levels of LSD1 and phosphorylated and total ERKs were analyzed by Western blot after LSD1 shRNA treatment. Actin was used as an internal control to verify equal protein loading. **b** Effects of the expression of control shRNA and LSD1 shRNA during pancreatic progenitor differentiation on NKX6.1 and CHGA expression and quantification of the proportion of NKX6.1^−^/CHGA ^+^ (**c**) cells from **b** as assayed at the end of stage 3. **d** The protein levels of phosphorylated and total ERKs were analyzed by Western blot after TCP treatment. Flow cytometry analysis of NGN3 expression at stage 3 (**e**) and quantification of the proportion of NGN3^+^ cells (**f**) from **e**. Flow cytometry analysis of NKX6.1 and the proliferation marker Ki67 in control and TCP-treated pancreatic progenitors (**g**) and quantification of co-expression of PDX1 and Ki67 in PP2 cells (**h**) from **g** as assayed at the end of stage 3. Flow cytometry analysis of PDX1 and the proliferation marker Ki67 in control and TCP-treated pancreatic progenitors (**i**), and quantification of co-expression of PDX1 and Ki67 in PP2 cells (**j**) from **i** as assayed at the end of stage 3. Data represent mean ± SEM, *p < 0.05, **p < 0.01, ***p < 0.001, two-sided student’s *t* test (*n* = 3 biologically independent samples per group)

To investigate the possible mechanism of LSD1 on IPC differentiation of hESCs, signaling pathways proteins of ES, DE, and PP2 were checked by Western blot (Fig. S2). Both p38 and ERK signaling were continuously activated during ES to PP2. LSD1 and total ERK protein were greatly reduced in LSD1-shRNA-treated group compared to control group, whereas phosphorylated ERKs were significantly increased (Fig. 4a). We observed a sharp increase of CHGA^+^/NKX6.1^−^ endocrine cells in cultures of PP2 cells treated with LSD1 shRNA (Fig. 4b, c). In agreement with the finding from LSD1 silencing, inhibition of LSD1 by TCP in stage 3 cells also leads to increased phosphorylated ERKs and decreased ERK (Fig. 4d). Then, we tested the effect of LSD1 inhibition during stage 2 to stage 3 of the IPC differentiation protocol. When LSD1 was inhibited by TCP during stage 3, cells differentiated into NGN3 ^+^ endocrine progenitors more efficiently than controls with 19.1 ± 3.1% TCP treatment vs. 11.2 ± 3.2% control (Fig. 4e, f). TCP treatment also results in a twofold increase in the proportion of PP2 cells co-expressing the proliferation marker Ki67 and NKX6.1 (Fig. 4g, h). On the contrary, cells co-expressing Ki67 and PDX1 drastically reduced from 66.2 to 19.1% (Fig. 4i, j). Inactivation of LSD1 by TCP or silencing LSD1 activated ERK signaling, leading to elevated proportion of NKX6.1^+^ PP2 cells.

To verify our hypothesis that LSD1 regulates ERK signaling during PP2 specification, we added both of LSD1-shRNA and ERK inhibitor PD98059 in the cell differentiation medium. Firstly, LSD1 knockdown increased p-ERK expression drastically, while PD98059 largely reduced p-ERK expression (Fig. S4.a). LSD1-shRNA and PD98059 treatment together brought p-ERK back to the normal level. In line with p-ERK expression, the proportion of PDX1^+^/NKX6.1^+^ cells was raised from 53.24 to 73.12% with LSD1-shRNA treatment, and PD98059 treatment drastically decreased the number of PDX1^+^/NKX6.1^+^ cells, to 22.81% (Fig. S4.b, c). Knockdown LSD1 in PD98059 group upregulated the ratio of PDX1^+^/NKX6.1^+^ cells, back to 46.76%, which was almost similar to that of the blank group (Fig. S4.b, c). Thus, both LSD1 and ERK signaling played vital role in PP2 specification. LSD1 promotes cell differentiation from DE to PP2 through ERK signaling activation.

### Sustained inhibition of LSD1 enhances functional β-cell production

**Fig. 5:**
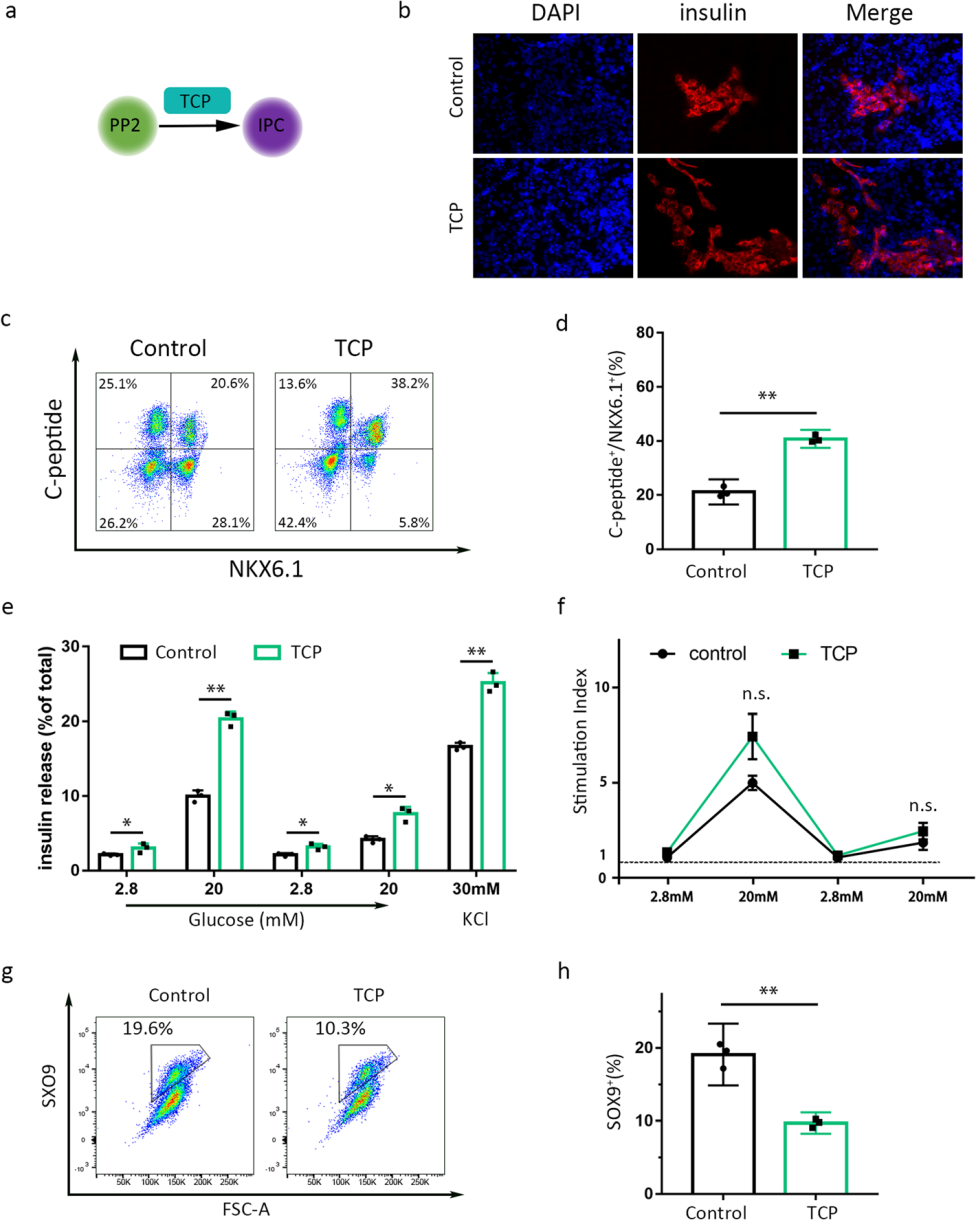
Functionality of ES-derived insulin-producing cells upon LSD1 inhibition. **a** Experimental design of the figure. **b** Representative immunofluorescent staining of insulin-producing cells for dapi (blue), insulin (red) in control, and LSD1 shRNA groups. **c** Flow cytometry of C-peptide and NKX6.1 expression in control and TCP groups and **d** proportion of cells co-expressing both markers at the end of differentiation. **e** Insulin secretion levels for control or TCP-treated stem cell-derived β cells during sequential rounds of glucose and KCl challenge. Insulin secretion levels were normalized to total insulin content for each sample. Stimulation indexes were calculated as a ratio of insulin secretion at high glucose (20 mM) relative to the basal secretion (2.8 mM glucose). **e** Flow cytometry of SOX9 expression in the control and TCP-treated differentiating progenitors and **f** quantification of the ratio of SOX9^+^ cells at the end of stage 4. Data represent mean ± SEM, *p < 0.05, **p < 0.01, ***p < 0.001, two-sided student’s *t* test (*n* = 3 biologically independent samples per group)

We continuously used TCP to inhibit LSD1 activity from stage 3 to stage 4 (Fig. 5a). By the end of the differentiation, we harvested the cells named IPC. Immunostaining results show a dramatic increase in the proportion of IPCs expressing insulin (Fig. 5b) in the TCP-treated group. In accordance with the finding, the proportion of cells co-expressing NKX6.1 and c-peptide nearly doubled in the TCP group compared with those of control group, 20.6% vs 38.2% (Fig. 5b, c). As known to all, functional pancreatic β cells can secrete insulin in response to glucose. In our study, IPCs of either control or TCP group during stages 4 of differentiation (Fig. 5a) show an increase in insulin secretion at high glucose over low glucose in response to sequential glucose stimulations (Fig. 5e, f). Moreover, the levels of insulin secretion per total insulin content at 2.8 mM glucose, 20 mM glucose, and 30 mM KCl were significantly higher in cultures of IPCs differentiated with TCP treatment (Fig. 5e). However, there were no statistically significant differences in stimulation indexes between control and TCP-treated IPCs (Fig. 5f). TCP treatment during the PP2 to IPC differentiation results in a double decrease in the proportion of SOX9^+^ ductal-like progenitor cells (Fig. 5g, h), which could partially explain where the increased IPCs come from.

## Discussion

Transplantation of insulin-producing cells derived from human embryonic stem cells (hESCs) has been proposed as a promising therapy for diabetes [23]. So the problem comes to generating pancreatic beta cells with better maturation in a large scale to substitute impaired islet. In this study, we adopted a modified four-step differentiation protocol to generate insulin-producing cells in vitro. We successfully induced human embryonic stem cell (H9) into functional IPCs. Campbell and Hoffman reviewed that chromatin regulators including histone methyltransferases could become promising targets for pancreas development and the maintenance of mature β cell function [13]. Based on the knowledge that histone demethylase LSD1 plays an important role in balancing the self-renewal and differentiation of hESCs/hiPSCs [15], regulating differentiation onset of trophoblast stem cells [18], and LSD1 with 50% activity drives stem cells to differentiation [16], we hypothesize that LSD1 may affect pancreatic differentiation from hESCs.

We performed loss-of-function experiments to explore the role of LSD1 in differentiation stages form hESCs to IPCs. Inactivation of LSD1 was achieved by shRNA lentivirus or inhibitor TCP treatment. For definitive endoderm specification, inhibition or knockdown LSD1 does not affect definitive endoderm commitment (Fig. 2, Fig. S3). We did supplementary experiments to monitor the dynamic change of Stage 1 differentiation and provided a complete picture of DE differentiation (Fig. S3). We employed immunofluorescence assay and flow cytometry to examine the proportion of SOX17^+^/CXCR4^+^ cells and SOX17^+^/FOXA2^+^ cells day by day. In fact, there was significant difference in definitive endoderm differentiation efficiency between control group and LSD1 knockdown group, with SOX17^+^/CXCR4^+^ cells ratio 22% vs 51%, 40% vs 61% in the first 2 days. But the difference was gone over time. The two groups obtained nearly 90% of SOX17^+^/CXCR4^+^ cells at the end of DE differentiation. We guess it is the modified induction protocol for DE differentiation used in this study that made the vast majority of hESCs turned into DE cells, regardless of LSD1 expression or activity. Nevertheless, LSD1 inhibition accelerated the differentiation from stem cells into DE cells at the beginning. It might be attributed to the fact that LSD1 inhibition help initiating stem cell differentiation at the very beginning, but we examine the definitive endoderm cells by the end of stage 1. As for pancreatic progenitor differentiation, we surprisingly discovered that a downregulated activity of LSD1, either chemically or genetically, enhances the generation of pancreatic progenitor cells and later improves IPCs maturation.

A great deal of effort has been made to optimize the timing of the activation or inhibition of key signaling pathways to obtain higher differentiation efficiency and better maturation of IPCs derived from stem cells. The Erk/MAPK pathway is known to be important for ESCs differentiation. Kim et al. demonstrated that ERK-mediated Nanog phosphorylation plays an important role in self-renewal of ES cells through FBXW8-mediated Nanog protein stability [24]. Li et al. reported identification of a target gene of Oct4, serine/threonine kinase 40 (Stk40), which is able to activate the Erk/MAPK pathway and induce extra embryonic endoderm differentiation in mouse ESCs [25]. Besides, Wnt signaling inhibition in vitro causes an increase in the proportion of differentiated endocrine cells [26]. Moreover, deep involvement of Notch signaling in the development of the pancreas and the lateral inhibition of Notch signaling in pancreatic progenitor differentiation and maintenance [27]. Functional experiments showed that activin /TGF-β signaling achieves this essential function by controlling the levels of transcription factors necessary for pancreatic development [28]. These findings indicate that the in vitro differentiation programs recapitulate multiple signaling pathways involved in embryonic pancreas development. In this regard, we provide the first demonstration of an association between LSD1 and the induced differentiation of insulin-producing cells from hESCs. During IPCs differentiation from hESCs, ERK, and p38/MAPK signaling were constantly activated (Fig. S2). Moreover inactivating LSD1 boosted ERK signaling activation, resulting increased production of pancreatic progenitors. This finding helps to optimize protocols to obtain more pancreatic progenitor cells from hESCs.

Human embryonic stem cell-derived NKX6.1-expressing pancreatic progenitor cells accelerate the maturation of insulin-secreting cells in vivo [29]. Transcription factor NKX 6.1 controls a gene regulatory network required for establishing and maintaining pancreatic β cell identity [30, 31]. The activation of endocrine differentiation in PDX1+/NKX6.1+ pancreatic progenitors finally produces subsequent glucose-responsive β-like cells in vitro [11, 32]. In our study, we observed that LSD1 inhibition lead to higher production of PDX1^+^/NKX6.1^+^ pancreatic progenitors. NKX6.1^+^ population shows increased proliferation ability with the treatment of TCP, while PDX1^+^ population got lower proliferation ability. These results might suggest that TCP treatment results in augmented expression of NKX6.1 in pancreatic progenitors, preparing for better maturation of insulin-producing cells in the last stage. Besides, LSD1 knockdown caused a sharp increase of CHGA^+^/NKX6.1^−^ population, which indicate pre-maturation of pancreatic progenitors [33].

Many studies revealed the vital role of transcription factor SOX9 in transition of pancreatic progenitor cells to mature pancreatic β cells [34–37]. Xu et al. reported that microRNA-690 regulates induced pluripotent stem cells (iPSCs) differentiation into insulin-producing cells by targeting Sox9 [35]. In concern with this finding, our data revealed that inhibition of LSD1 upregulated SOX9 expression in stage 3, accompanied by higher ratio of pancreatic progenitors (Fig. 3b). On the contrary, Sox9 was largely reduced in stage 4, indirectly helping the maturation of insulin-producing cells (Fig. 5g, h). It might suggest that more pancreatic progenitor cells become IPCs with treatment of TCP due to reduced expression of SOX9 in the last differentiation population. In this paper, we identify LSD1, one of the regulators of the ERK signaling pathway, as a factor involved in pancreatic progenitor specification and differentiation into functional insulin-producing cells. However, this finding was only obtained in human embryonic stem cell line H9; it would be of great significance if researchers attempt to apply this protocol to other cell lines.

## Conclusions

In summary, our study demonstrates that knockdown or inhibition of LSD1 promoted pancreatic progenitors differentiation of hESCs through activating ERK signaling, and sustained TCP treatment improved the IPCs maturation witnessed by better GSIS. Therefore, our data suggest that inhibition of LSD1 by TCP is expected to optimize differentiation protocols for mature pancreatic β-like cells from human embryonic stem cells and would push one step forward to apply cell-based therapies for diabetes.

## Supplementary information

**Additional file 1: Figure S1.** LSD1 knockdown by shRNA lentivirus and its inhibitor TCP. (a) LSD1 knock down causes morphological changes in H9 cells. Morphology of H9 colonies transduced with a control short hairpin (control), the short hairpin 1 (sh1), the short hairpin 2 (sh2), and the short hairpin 3 (sh3) against LSD1. Upper panels show cell images and lower panels show the expression of GFP by fluorescence. (b) Inhibitory effects of an LSD1 inhibitor TCP at different concentrations detected by the Epigenase™ LSD1 demethylase Activity/Inhibition Assay Kit (Fluorometric). (c) LSD1 activity in 40 μM TCP treated group and control group.

**Additional file 2: Figure S2.** Representative images of signaling pathways checked by western blot in hESCs (H9), definitive endoderm cells (DE) and PDX1^+^/NKX6.1^+^pancreatic progenitors cells (PP2).

**Additional file 3: Figure S3.** A complete picture of definitive endoderm differentiation in LSD1 knockdown group and control group. (a) SOX17^+^/CXCR4^+^ cells was examined by flow cytometry assay every day during DE differentiation. (b) SOX17^+^/FOXA2^+^ cells was checked by immunofluorescence assay each day during DE differentiation.

**Additional file 4: Figure S4.** Knocking-down LSD1 activated ERK signaling and promotes PP2 specification. (a) ERK signaling was activated by LSD1-shRNA treatment and was blocked by ERK inhibitor PD98059 treatment of pancreatic progenitor (PP2) cells as assessed by immunoblot analysis with anti-phospho-ERK, ERK, LSD1 and Actin antibodies. (b) The co-expression of PDX1 with NKX6.1 were detected by immunofluorescence assay in with the treatment of LSD1-shRNA, PD98059, and both at the differentiation stage 3 respectively. (c) The co-expression of PDX1 and NKX6.1 during pancreatic progenitor differentiation was assessed by flow cytometry in the four groups and the proportion of PDX1^+^/NKX6.1^+^ cells was shown in the scatter diagram respectively.

**Additional file 5: Table S1.** Information about LSD1 shRNAs.

**Additional file 6: Table S2.** Primer sequences for real time PCR.

**Additional file 7: Table S3.** Antibodies used in this study.

## Data Availability

Data supporting our findings can be found in the additional files. We also welcome emails to discuss any interested questions related to this paper.

## Abbreviations

hESCs: Human embryonic stem cells
hiPSCs: Human induced pluripotent stem cells
IPCs: Insulin-producing cells
GSIS: Glucose-stimulated insulin secretion
LSD1: Lysine-specific demethylase 1
mESCs: Mouse embryonic stem cells
iPSCs: Induced pluripotent stem cells
TCP: Tranylcypromine
TSCs: Trophoblast stem cells
PDX1: Pancreatic And Duodenal Homeobox 1
NKX6.1: NK homeobox factor 6.1
NGN3: Neurogenin-3
qPCR: Quantitative real-time reverse transcription polymerase chain reaction
SDS-PAGE: Sodium dodecyl sulfate-polyacrylamide gel electrophoresis
PVDF: Polyvinylidene difluoride
