# Supplementary figures and images for: LSD1 inhibition yields functional insulin-producing cells from human embryonic stem cells
Source: Stem Cell Res Ther. 2020 Apr 28;11:163. doi: 10.1186/s13287-020-01674-y (PMC7189473; doi:10.1186/s13287-020-01674-y)

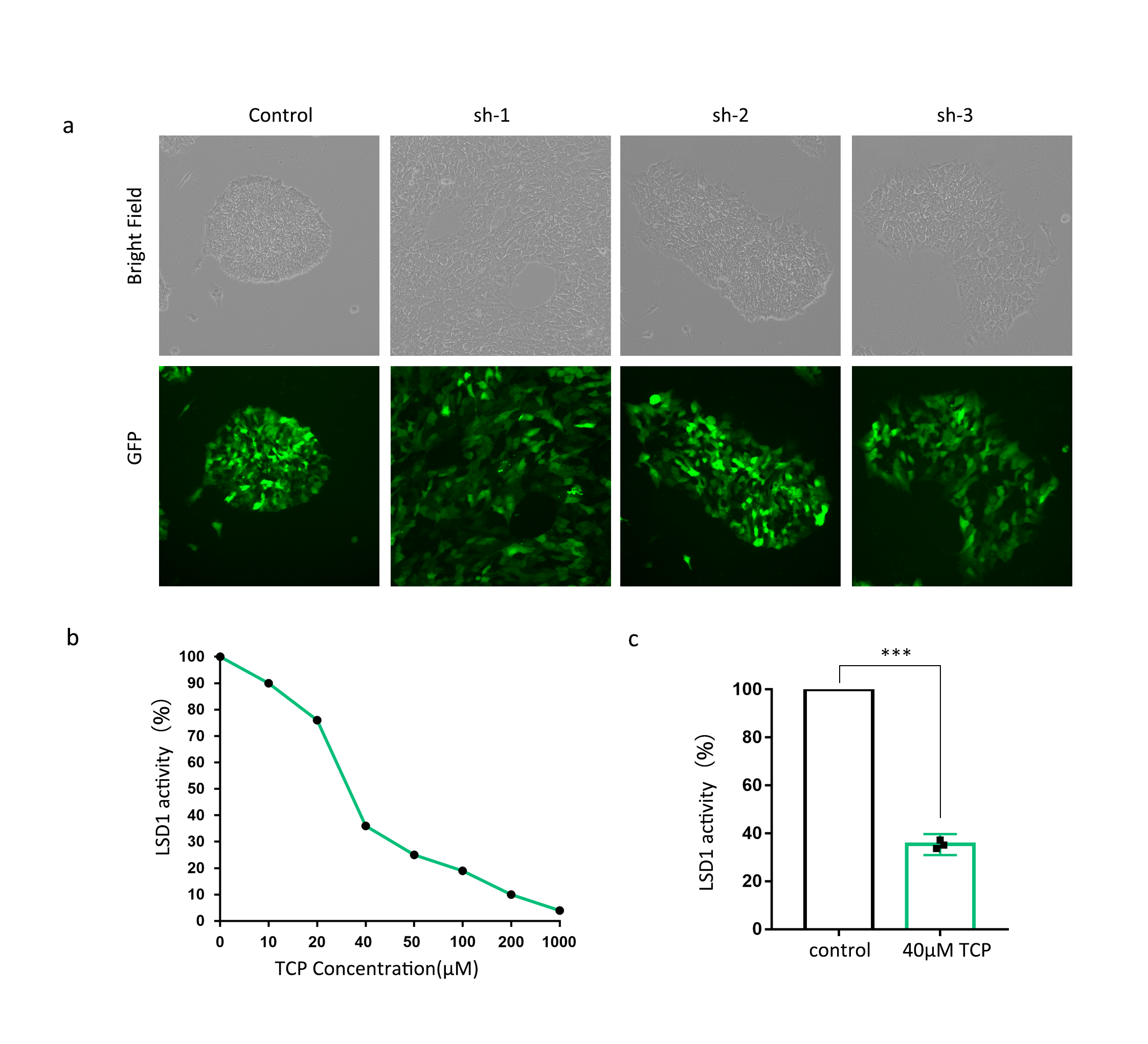

Supplement: Supplementary file 1 — Additional file 1: Figure S1. LSD1 knockdown by shRNA lentivirus and its inhibitor TCP. (a) LSD1 knock down causes morphological changes in H9 cells. Morphology of H9 colonies transduced with a control short hairpin (control), the short hairpin 1 (sh1), the short hairpin 2 (sh2), and the short hairpin 3 (sh3) against LSD1. Upper panels show cell images and lower panels show the expression of GFP by fluorescence. (b) Inhibitory effects of an LSD1 inhibitor TCP at different concentrations detected by the Epigenase™ LSD1 demethylase Activity/Inhibition Assay Kit (Fluorometric). (c) LSD1 activity in 40 μM TCP treated group and control group. [file 13287_2020_1674_MOESM1_ESM.tif]

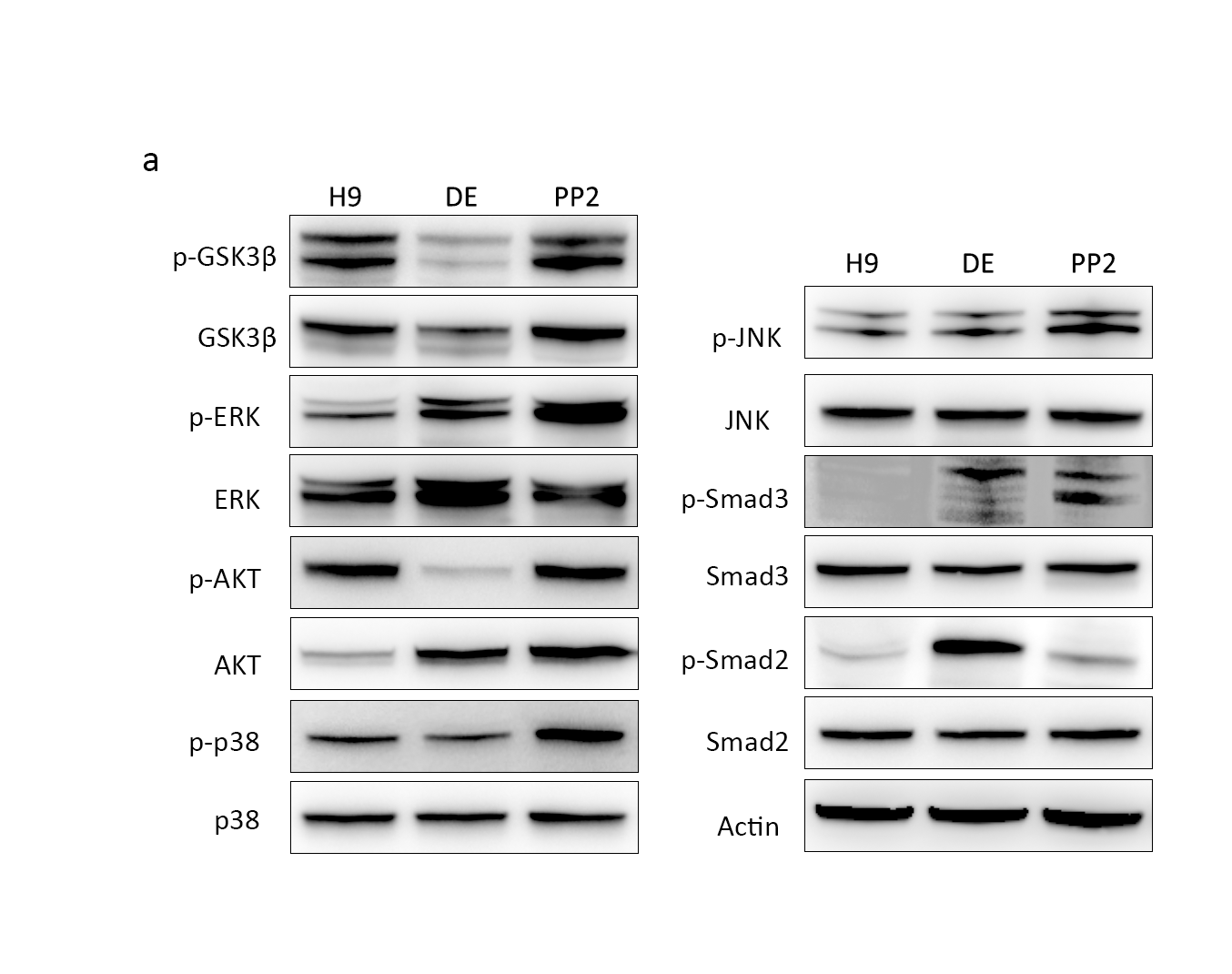

Supplement: Supplementary file 2 — Additional file 2: Figure S2. Representative images of signaling pathways checked by western blot in hESCs (H9), definitive endoderm cells (DE) and PDX1+/NKX6.1+pancreatic progenitors cells (PP2). [file 13287_2020_1674_MOESM2_ESM.tif]

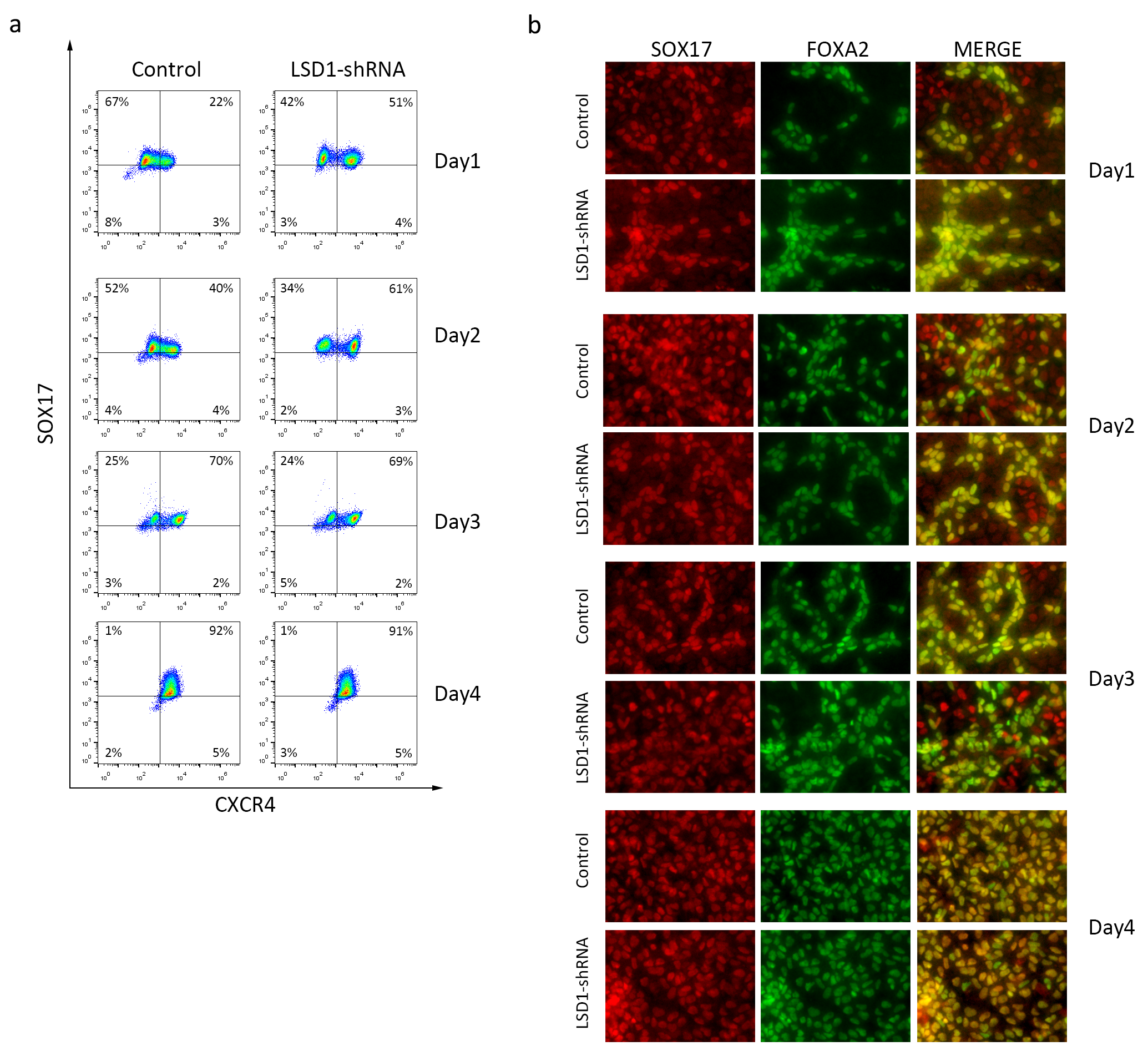

Supplement: Supplementary file 3 — Additional file 3: Figure S3. A complete picture of definitive endoderm differentiation in LSD1 knockdown group and control group. (a) SOX17+/CXCR4+ cells was examined by flow cytometry assay every day during DE differentiation. (b) SOX17+/FOXA2+ cells was checked by immunofluorescence assay each day during DE differentiation. [file 13287_2020_1674_MOESM3_ESM.tif]

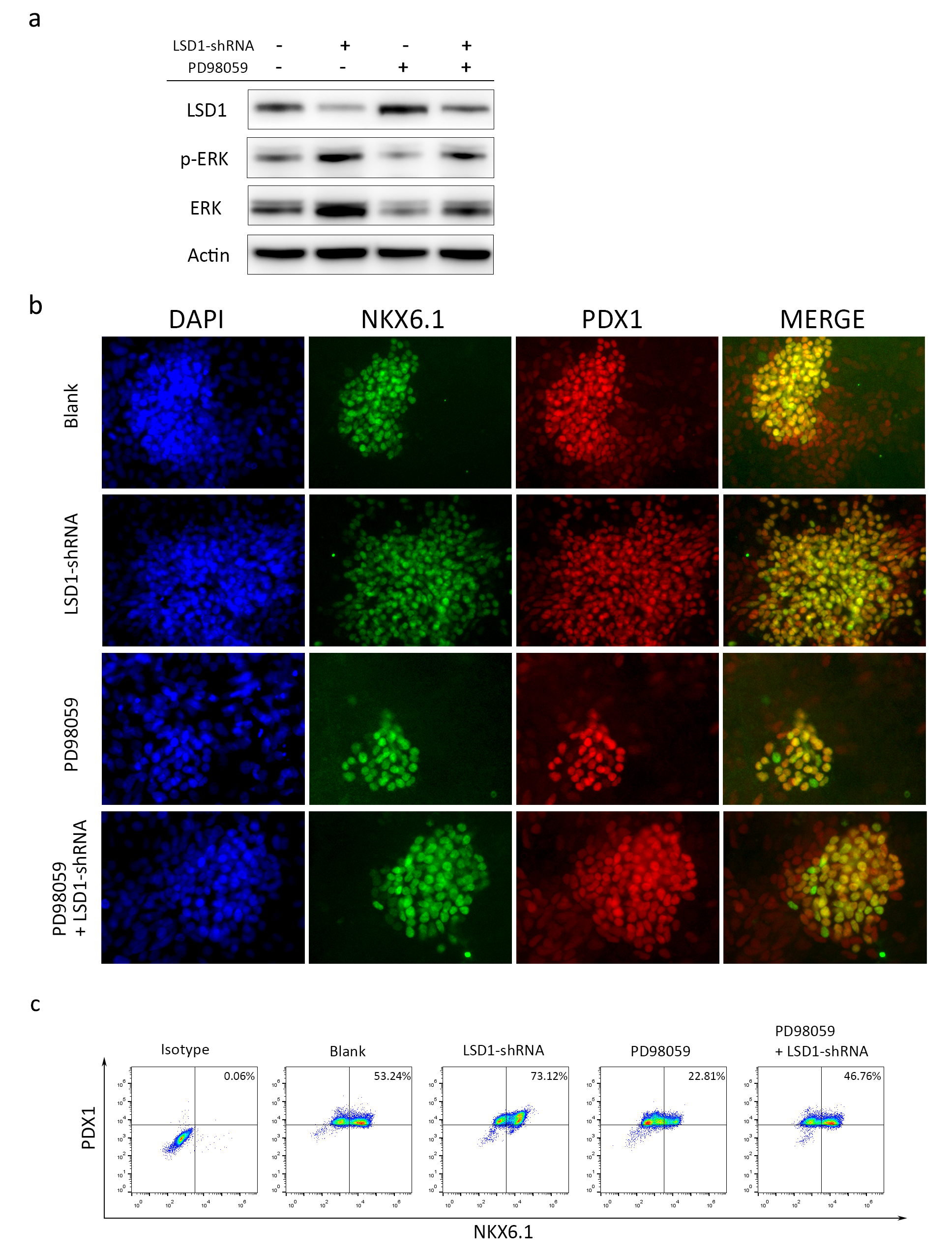

Supplement: Supplementary file 4 — Additional file 4: Figure S4. Knocking-down LSD1 activated ERK signaling and promotes PP2 specification. (a) ERK signaling was activated by LSD1-shRNA treatment and was blocked by ERK inhibitor PD98059 treatment of pancreatic progenitor (PP2) cells as assessed by immunoblot analysis with anti-phospho-ERK, ERK, LSD1 and Actin antibodies. (b) The co-expression of PDX1 with NKX6.1 were detected by immunofluorescence assay in with the treatment of LSD1-shRNA, PD98059, and both at the differentiation stage 3 respectively. (c) The co-expression of PDX1 and NKX6.1 during pancreatic progenitor differentiation was assessed by flow cytometry in the four groups and the proportion of PDX1+/NKX6.1+ cells was shown in the scatter diagram respectively. [file 13287_2020_1674_MOESM4_ESM.tif]
