# Supplementary material for: LSD1 inhibition yields functional insulin-producing cells from human embryonic stem cells
Source: Stem Cell Res Ther. 2020 Apr 28;11:163. doi: 10.1186/s13287-020-01674-y (PMC7189473; doi:10.1186/s13287-020-01674-y)
Supplement: Supplementary file 5 — Additional file 5: Table S1. Information about LSD1 shRNAs. [file 13287_2020_1674_MOESM5_ESM.docx]

**Table S1** information about LSD1 shRNAs.

**Vector**：GV248

**Main component of the vector**：hU6-MCS-Ubiquitin-EGFP-IRES-puromycin

**Control nonsense sequence**: TTCTCCGAACGTGTCACGT

| **ID** | **5’** | **stem** | **loop** | **stem** | **3’** |
| --- | --- | --- | --- | --- | --- |
| LSD1-sh1-a | Ccgg | CGGACAAGCTGTTCCTAAA | CTCGAG | TTTAGGAACAGCTTGTCCG | TTTTTg |
| LSD1-sh1-b | aattcaaaaa | CGGACAAGCTGTTCCTAAA | CTCGAG | TTTAGGAACAGCTTGTCCG |  |
| LSD1-sh2-a | Ccgg | GAACTCCATCAGCAATACA | CTCGAG | TGTATTGCTGATGGAGTTC | TTTTTg |
| LSD1-sh2-b | aattcaaaaa | GAACTCCATCAGCAATACA | CTCGAG | TGTATTGCTGATGGAGTTC |  |
| LSD1-sh3-a | Ccgg | CACAAGGAAAGCTAGAAGA | CTCGAG | TCTTCTAGCTTTCCTTGTG | TTTTTg |
| LSD1-sh3-b | aattcaaaaa | CACAAGGAAAGCTAGAAGA | CTCGAG | TCTTCTAGCTTTCCTTGTG |  |
