# Supplementary material for: LSD1 inhibition yields functional insulin-producing cells from human embryonic stem cells
Source: Stem Cell Res Ther. 2020 Apr 28;11:163. doi: 10.1186/s13287-020-01674-y (PMC7189473; doi:10.1186/s13287-020-01674-y)
Supplement: Supplementary file 6 — Additional file 6: Table S2. Primer sequences for real time PCR. [file 13287_2020_1674_MOESM6_ESM.docx]

**Table S2** Primer sequences for real time PCR.

| Gene | Primer sequence | |
| --- | --- | --- |
| ACTB | Forward | 5’-CATGTACGTTGCTATCCAGGC-3’ |
|  | Reverse | 5’-CTCCTTAATGTCACGCACGAT-3’ |
| LSD1 | Forward | 5’-TCCTGGCCCCTCGATTC-3’ |
|  | Reverse | 5’-ATGTTCTCCCGCAAAGAAGAGT-3’ |
| OCT4 | Forward | 5’GGAGGAAGCTGACAACAATGAAA-3’ |
|  | Reverse | 5’-GGCCTGCACGAGGGTTT-3’ |
| SOX2 | Forward | 5’-TGCGAGCGCTGCACAT-3’ |
|  | Reverse | 5’-TCATGAGCGTCTTGGTTTTCC-3’ |
| NANOG | Forward | 5’-ACAACTGGCCGAAGAATAGCA-3’ |
|  | Reverse | 5’-GGTTCCCAGTCGGGTTCAC-3’ |
| SXO17 | Forward | 5’-TGGCGCAGCAGAATCCA-3’ |
|  | Reverse | 5’-CCACGACTTGCCCAGCAT-3’ |
| CXCR4 | Forward | 5’-CACCGCATCTGGAGAACCA-3’ |
|  | Reverse | 5’-GCCCATTTCCTCGGTGTAGTT-3’ |
| FOXA2 | Forward | 5’-GGGAGCGGTGAAGATGGA-3’ |
|  | Reverse | 5’-TCATGTTGCTCACGGAGGAGTA-3’ |
| PDX1 | Forward | 5’-TGGAGCTGGCTGTCATGTTGA-3’ |
|  | Reverse | 5’-CGCTTCTTGTCCTCCTCCTTTT-3’ |
| NKX6.1 | Forward | 5’-CTGGCCTGTACCCCTCATCA-3’ |
|  | Reverse | 5’-CTTCCCGTCTTTGTCCAACAA-3’ |
| PAX4 | Forward | 5’-ACTGTATGGCTTGGAATGAGG-3’ |
|  | Reverse | 5’-CAGGACGGTAAGGACAATGG-3’ |
| PAX6 | Forward | 5’-GCTTCACCATGGCAAATAACC-3’ |
|  | Reverse | 5’-GGCAGCATGCAGGAGTATGA-3’ |
| NGN3 | Forward | 5’-CTATTCTTTTGCGCCGGTAG-3’ |
|  | Reverse | 5’-ACTTCGTCTTCCGAGGCTCT-3’ |
| NEUROD1 | Forward | 5’-GGATGACGATCAAAAGCCCAA-3’ |
|  | Reverse | 5’-GCGTCTTAGAATAGCAAGGCA-3’ |
| SOX9 | Forward | 5’-AGCTCTGGAGACTTCTGAACGAGAG-3’ |
|  | Reverse | 5’-CGTTCTTCACCGACTTCCTCCGC-3’ |
| NKX2.2 | Forward | 5’-ATGTAAACGTTCTGACAACT-3’ |
|  | Reverse | 5’-TTCCATATTTGAGAAATGTTTGC-3’ |
