# Supplementary material for: LSD1 inhibition yields functional insulin-producing cells from human embryonic stem cells
Source: Stem Cell Res Ther. 2020 Apr 28;11:163. doi: 10.1186/s13287-020-01674-y (PMC7189473; doi:10.1186/s13287-020-01674-y)
Supplement: Supplementary file 7 — Additional file 7: Table S3. Antibodies used in this study. [file 13287_2020_1674_MOESM7_ESM.docx]

**Table 3** Antibodies used in this study.

| **Antibodies** | **Source** | **Cat.NO** | **usage** |
| --- | --- | --- | --- |
| Rabbit anti-p53 | Cell Signaling Technology | 9284S | WB |
| Rabbit anti-JNK | Cell Signaling Technology | 9252S | WB |
| Rabbit anti-p-JNK | Cell Signaling Technology | 9251S | WB |
| Rabbit anti-AKT | Cell Signaling Technology | 9272S | WB |
| Rabbit anti-p-AKT | Cell Signaling Technology | 9271S | WB |
| Rabbit anti-ERK | Cell Signaling Technology | 4695S | WB |
| Rabbit anti-p-ERK | Cell Signaling Technology | 4370S | WB |
| Rabbit anti-p38 | Cell Signaling Technology | 9212S | WB |
| Rabbit anti-p-p38 | Cell Signaling Technology | 9211S | WB |
| Rabbit anti-GSK3β | Cell Signaling Technology | 9315S | WB |
| Rabbit anti-p-GSK3β | Cell Signaling Technology | 9331S | WB |
| Mouse anti-Smad2 | Cell Signaling Technology | 3103S | WB |
| Rabbit anti-p-Smad2 | Cell Signaling Technology | 3108S | WB |
| Rabbit anti-Smad3 | Cell Signaling Technology | 9523S | WB |
| Rabbit anti-p-Smad3 | Cell Signaling Technology | 9520S | WB |
| Rabbit anti-LSD1 | Cell Signaling Technology | 2139 | WB, IF |
| Rabbit anti-OCT4 | Abcam | ab181557 | WB,IF |
| Rabbit anti-Nanog | Abcam | Ab80892 | WB |
| Mouse anti-β-Actin | Transgen | HC201-02 | WB |
| Mouse anti-SOX17 | Abcam | Ab84990 | IF |
| Rabbit anti-FOXA2 | Cell Signaling Technology | 8186 | IF |
| SOX17-(APC) | R&D | IC1924A | FC |
| CXCR4-(Fluorescein) | R&D | FAB170F | FC |
| Rabbit anti-PDX1 | Abcam | ab47267 | IF |
| Rabbit anti-NKX6.1 | DSHB | F55A12-c | IF |
| PDX1-(Fluorescein) | BD | 562274 | FC |
| NKX6.1-(APC) | BD | 563338 | FC |
| Guinea pig anti-insulin | Abcam | Ab7842 | IF |
| Insulin-(APC) | R&D | 8580S | FC |
| c-Peptide | DSHB | GN-ID4-c | FC |
|  |  |  |  |
|  |  |  |  |
|  |  |  |  |
|  |  |  |  |
|  |  |  |  |
|  |  |  |  |

FC, Flow cytometry; IF, Immunofluorescence assay; WB, western blot.
